# Utilization of patterned bioprinting for heterogeneous and physiologically representative reconstructed epidermal skin models

**DOI:** 10.1038/s41598-021-85553-3

**Published:** 2021-03-18

**Authors:** Sabrina Madiedo-Podvrsan, Jean-Philippe Belaïdi, Stephanie Desbouis, Lucie Simonetti, Youcef Ben-Khalifa, Christine Collin-Djangone, Jérémie Soeur, Maïté Rielland

**Affiliations:** L’Oréal Research and Innovation, Aulnay-sous-Bois, France

**Keywords:** Cell biology, Skin diseases, Tissue engineering

## Abstract

Organotypic skin tissue models have decades of use for basic research applications, the treatment of burns, and for efficacy/safety evaluation studies. The complex and heterogeneous nature of native human skin however creates difficulties for the construction of physiologically comparable organotypic models. Within the present study, we utilized bioprinting technology for the controlled deposition of separate keratinocyte subpopulations to create a reconstructed epidermis with two distinct halves in a single insert, each comprised of a different keratinocyte sub-population, in order to better model heterogonous skin and reduce inter-sample variability. As an initial proof-of-concept, we created a patterned epidermal skin model using GPF positive and negative keratinocyte subpopulations, both printed into 2 halves of a reconstructed skin insert, demonstrating the feasibility of this approach. We then demonstrated the physiological relevance of this bioprinting technique by generating a heterogeneous model comprised of dual keratinocyte population with either normal or low filaggrin expression. The resultant model exhibited a well-organized epidermal structure with each half possessing the phenotypic characteristics of its constituent cells, indicative of a successful and stable tissue reconstruction. This patterned skin model aims to mimic the edge of lesions as seen in atopic dermatitis or ichthyosis vulgaris, while the use of two populations within a single insert allows for paired statistics in evaluation studies, likely increasing study statistical power and reducing the number of models required per study. This is the first report of human patterned epidermal model using a predefined bioprinted designs, and demonstrates the relevance of bioprinting to faithfully reproduce human skin microanatomy.

## Introduction

Human skin is highly heterogeneous with variability across individuals, anatomical sites and during skin pathology. A skin region can be of thickness ranging from 0.1 on the eye-lid up to more than 1 mm on the palm and sole, glabrous or hairy, and of different cell content and extra-cellular matrix (ECM) organization across different anatomical sites^1–3^*.* There are also dramatic interpersonal variations in pH, moisture, thickness, organization and microbiota. Furthermore, skin status is continually changing due to aging, UV exposure, pollution, environment, lifestyle or disease states^1^. Accurately modelling the physiology and the physiopathology of human skin requires in vitro organotypic models capable of reproducing key areas of diversity.

Skin was the first organ to be reconstructed in vitro by tissue engineering in 1981^4^, and has since been utilized for knowledge studies, the treatment of burns, scars and for plastic surgery^5^, and forms the backbone of efficacy and toxicity screening strategies used in the pharmaceutical and cosmetic industries^6–8^*.* Despite considerable advances in tissue engineering for skin, the organotypic model state-of-the-art remains far from reproducing the complexity of native human skin, resulting in limited predictability in research and development applications and evaluation tests^9,10^. While the need for more physiological skin models is clear, creating such models using conventional culture methods is highly challenging due to insufficient cell deposition precision to accurate recreate native human skin conditions.

Three-dimensional bioprinting, more commonly simply known as bioprinting, is an additive manufacturing technique that utilizes a printing system able to precisely deposit biological material such as cells and biocompatible matrices into complex and functional 3D architectures, resulting in organ reconstructions more reflective of human tissue than otherwise possible^11–13^. The accurate cell deposition available in bioprinting offers the possibility to reproduce skin heterogeneity, and therefore create more physiologically relevant skin models. This potential is exemplified by the work of Ng and colleagues, who developed a bioprinted pigmented full thickness skin model in which the melanocytes were spatially positioned to mimic pigmented blemishes^14^. Leveraging the spatial positioning accuracy of bioprinting to reproduce keratinocyte heterogeneity within in vitro skin models would allow for improved understanding skin physiology and accurate modelling of skin pathology such as that seen within photoaging, atopic dermatitis (AD) or ichthyosis vulgaris (IV)^15–17^*.* Furthermore, the ability to create a patterned reconstructed model of two separate skin phenotypes, for example one healthy and one pathological, within a single skin model insert may accurately reflect the heterogeneity of pathological skin, while also reducing inter-sample variability and improving the robustness of evaluation studies.

To validate the concept of patterned epidermal model, we first generated a proof-of-concept heterogeneous epidermal model using a normal human keratinocyte (NHK) population divided into GFP positive and negative subpopulations. We then extended this concept to a published physiologically relevant filaggrin down-regulated epidermal model, which mimics numerous defects observed in the epidermis of AD and IV patients, especially the drastic reduction of keratohyalin granules^18–20^. One-half of the reconstructed skin insert was reconstructed with shLuciferase-GFP transduced NHKs to function as a control section, with the other half of the insert reconstructed with fillagrin knockdown (shFLG) transduced NHKs.

This is the first report of a patterned bioprinting approach being used to reproduce the heterogeneity of the epidermis within an organotypic epidermal model, and represents a significant advancement in skin tissue engineering for both knowledge and evaluation studies.

## Results

### Lentiviral transduction and bioprinting of primary human keratinocytes does not impair epidermis reconstruction

**Figure 1:**
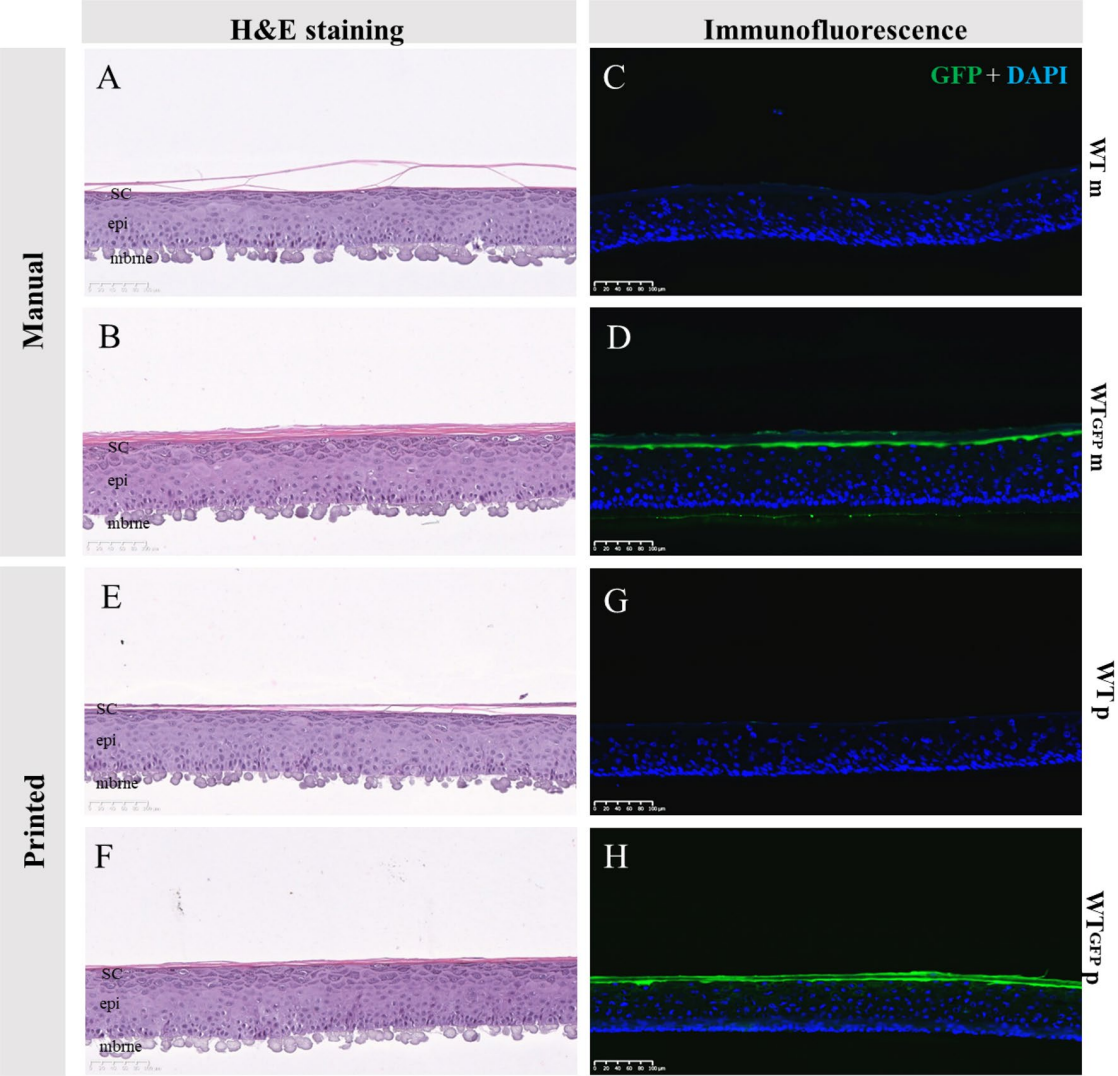
Effect of GFP transfection and bioprinting on epidermal reconstruction morphology. H&E morphology between skin models reconstructed from WT or WT-GFP cells was consistent for both manually (**A**,**B**) or bioprinted (**E**,**F**) samples. As anticipated, samples printed with WT-GFP (**D**,**H**) but not WT cells (**C**,**G**) express GPF, with no difference between those using manual (**C**,**D**) or (**G**,**H**) reconstruction techniques. *SC* stratum corneum, *epi* epidermis, *mbrne* polycarbonate membrane support, *WTm* manual reconstructed skin substitute from WT NHKs, *WTp* printed reconstructed skin substitute from WT NHKs, *WT-GFPm* manual reconstructed skin substitute from WT-GFP NHK, *WT-GFPm* printed reconstructed skin substitute from WT-GFP NHK (n = 6 for each condition).

As an initial proof of concept we utilized two populations of NHK; one of native cells (hereafter referred to as wild type (WT) cells), the second transfected with a lentiviral particle expressing Green Fluorescent Protein (WT-GFP) (Supplementary Fig. 1C,D). Both cell types demonstrated normal polygonal cell morphology in 2D monoculture (Supplementary Fig. 1A,B). The two groups were then either bioprinted or manually seeded to determine if inkjet bioprinting was influencing GFP expression or compromise the ability of the cells to form a reconstructed epidermis. Models using both manual and printed WT and WT-GFP keratinocytes demonstrated a well-organized basal layer, spinous layer, granular layer and stratum corneum (Fig. 1A,B,E,F).

GFP expression was particularly prominent in the granular layer and in the stratum corneum of the WT-GFP epidermis (Fig. 1D,H), but was still present in the basal and spinous layers, albeit at a lower intensity. As expected GFP expression was not detected in WT epidermis (Fig. 1C,G).

The expression and localization of key epidermal markers keratin 14 (K14, basal layer marker), keratin 10 (K10, spinous layer marker), and transglutaminase 1 (TGM1), loricrin (LOR) and filaggrin (FLG), three key markers of final differentiation, were all consistent with published reports^21,22^, with no difference between manual or printed epidermal models or WT and WT-GFP NHKs reconstructed models (Supplementary Fig. 2). These data demonstrate neither GFP transduction nor inkjet bioprinting hamper the ability of NEK to form a reconstructed epidermis consistent with native skin.

### Patterned human keratinocytes are stable in culture and form a fully differentiated patterned epidermis

**Figure 2:**
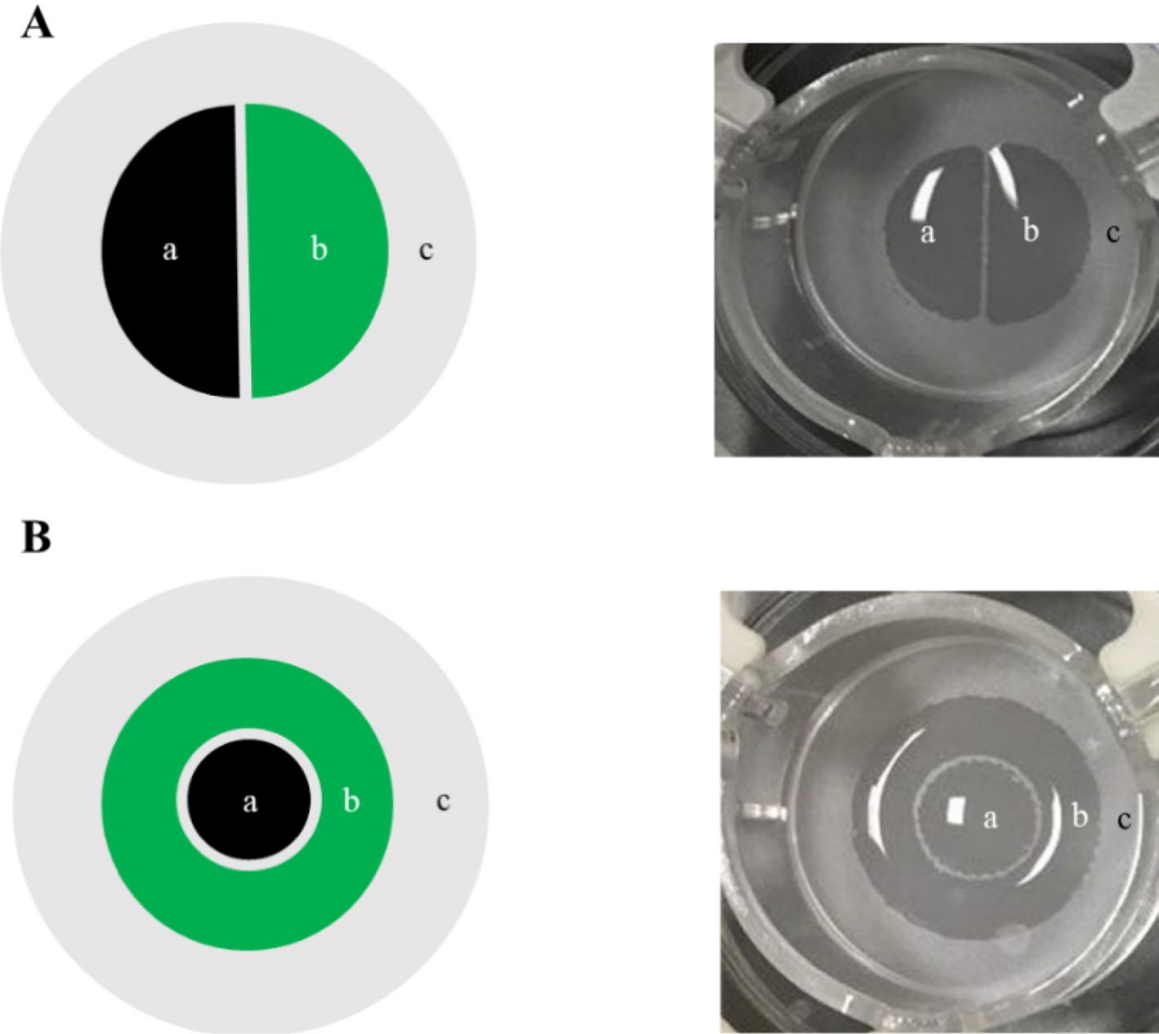
Macroscopic visualization of the cellular deposition design. Cells were printed in patterned cellular suspension in either semi-circles (**A**) or concentric rings (**B**) schematically illustrated as 2 separate NHK populations (left) or after seeding on a polycarbonate membrane after the bioprinting process (right). Pattern designs were drawn internally using BioCaD software (RegenHU).

We next adapted the bioprinting technique to obtain controlled and reproducible reconstructed epidermis patterns. We utilized two designs; semi-circles, which easily allow for half-and-half tissue collection, or concentric rings, which resemble a dermatological spot (Fig. 2A,B). Identifying the correct NHK concentrations with the appropriate media deposition is the most critical part of the process to obtain the optimal space between both compartments. If the cells are not sufficiently concentrated, the 3D models will not reconstruct correctly; if the cell concentration is too high clots may occur during printing and NHK mortality will increase (data not shown). After fixing the printing protocol, each compartment was separated by an empty space of 1.5 mm by using a hydrophobic membrane. We optimized of the quantity of liquid, number of cells and patterns of drop depositions to obtain an acceptable separation distance between compartments (Fig. 2A,B). The printing process took ~ 20 s per sample and was highly reproducible (data not shown).

**Figure 3:**
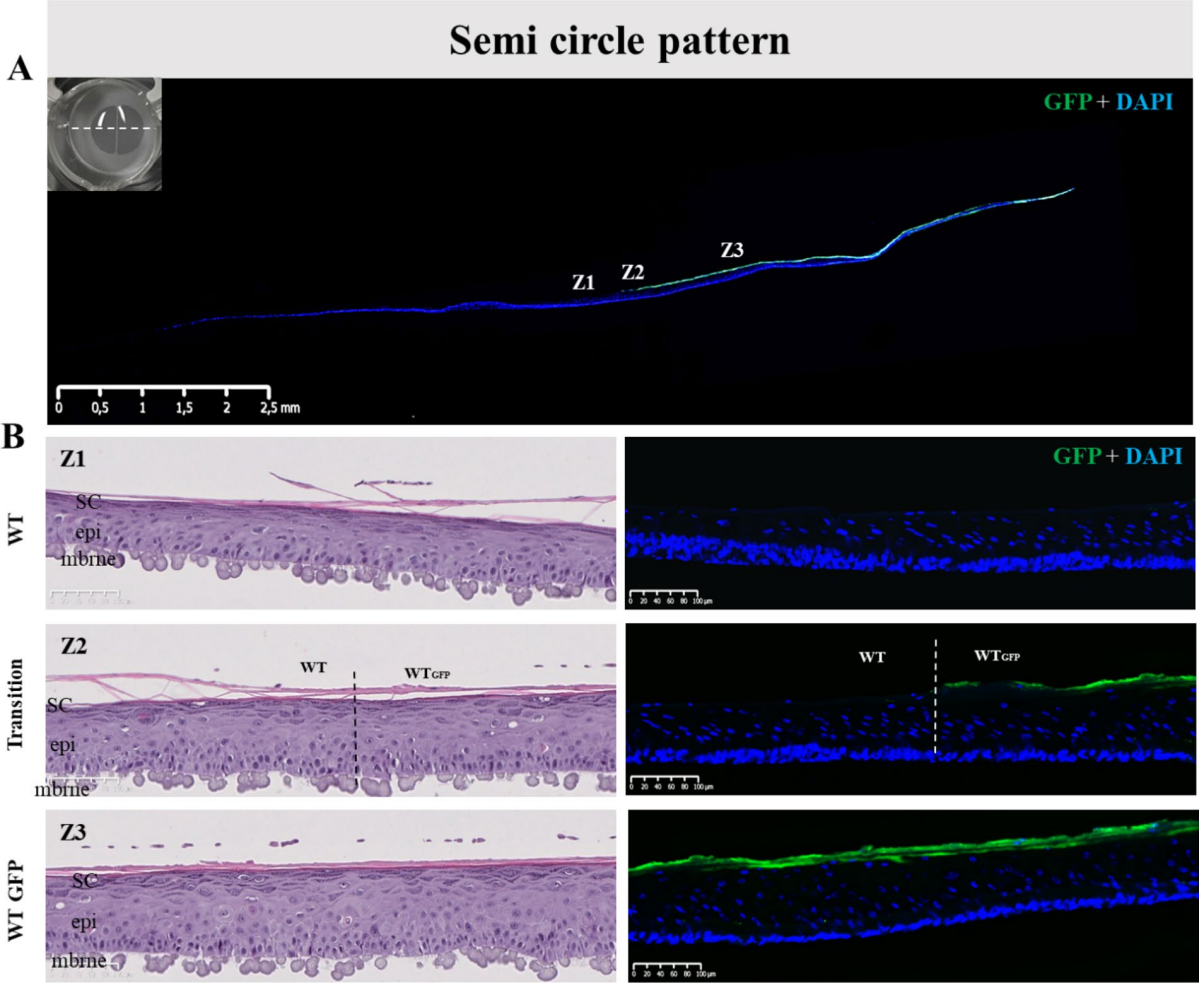
Histology of pattern bioprinted epidermis comprised of two distinct cell populations. (**A**) Lateral view of the reconstructed skin stained for GFP (green) and cell nuclei (DAPI, blue) at day 14 post-reconstruction. Indicated positions Z1–3 correspond to (**B**) H&E and immunofluorescence stained sections Z1–3, whereby Z1 is from the WT printed cell half, Z2 is the WT/WT-GFP cell interface, and Z3 is representative of the WT-GFP printed half. The H&E images (left) show no difference in gross morphology, while the immunofluorescence images (right) reveal no GFP staining on the WT cell side of the model, a clear demarcation where the WT and WT-GFP halves meet, and a consistent GFP signal in the WT-GFP printed half, demonstrating the model patterning to be stable across 14 days of culture. Images representative of 10 separate models.

**Figure 4:**
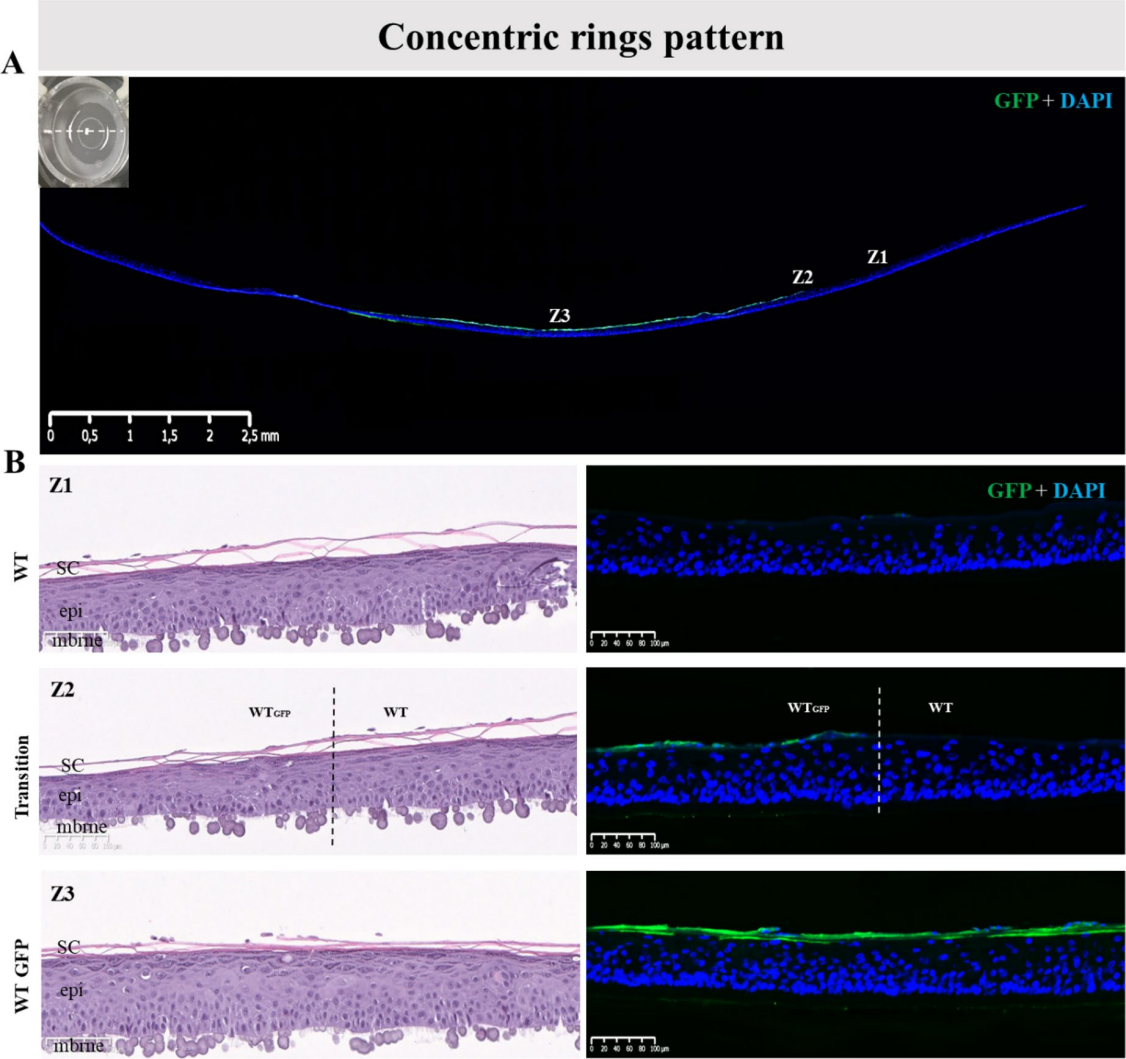
Bioprinted RHE constructs were achieved using distinct populations of NHKs on a same sample in order to pattern the concentric design. Morphological analysis of the printed epidermal substitutes and validation of the pattern using H&E staining and fluorescence analysis were done after 14 days of culture at air–liquid interface. (**A**) GFP staining (Green) on the entire skin substitute with nuclei counterstain with DAPI (blue) where we can observe both conserved parts of the epidermal sample, Z1, Z2 and Z3 are for zone 1, zone 2 and zone 3 represent the areas illustrated in (**B**); a macroscopic picture of the semi-circle sample has been represented with the orientation of sample slices for the analysis; (**B**) H&E staining and Green fluorescence analysis on the WT part of the sample (Z1), the transition between the WT and the WTGFP parts (Z2) and the WTGFP part (Z3) proving that pattern is conserved after 14 days of culture and that both NHK populations form at the end of the culture a unique sample. *Sc* stratum corneum, *epi* epidermis, *mbrne* polycarbonate membrane support, *WT* reconstructed epidermal part from native phenotypic keratinocytes, *WTGFP* reconstructed skin substitute from GFP-transduced native phenotypic keratinocytes, *Transition* specific region of the patterned reconstructed skin substitute where both WT and WTGFP are in contact (n = 11).

To examine if the printed NHK subpopulations are spatially stable within the sample we printed both WT and WT-GFP groups in semi-circle and the concentric circle depositions and examined the tissue histology and GFP distribution pattern (Figs. 3A,B, 4A,B). For both designs, the epidermis of printed models was fully differentiated with the presence of all epidermal layers (Figs. 3B-Z1, Z2 and Z3, 4B-Z1, Z2 and Z3). Histological analysis shows that the original gap between the patterned compartments to have been populated by cells and closed (Figs. 3A,B-Z2, 4A,B-Z2, Supplementary Fig. 3). Moreover, the clear demarcation of GFP expression shows that both compartments kept their original design with no merging of cell populations (Figs. 3A,B-Z2, 4A,B-Z3). We also found the expression of epidermal markers K14, K10, TGM1, LOR and FLG to be homogeneously expressed all along the samples, with no visible difference between WT and WT-GFP printed sections (Supplementary Fig. 4). These data demonstrate that NHK bioprinted patterning results in separate and stable populations without compromising reconstructed epidermis structure or morphology.

### Bioprinting of shLUC and shFLG human keratinocytes does not affect epidermis reconstruction

We next applied the pattern bioprinting technique to a more physio-pathologically relevant condition in order to create healthy “control” and “pathological” skin sections within the same sample, by utilizing a filaggrin (FLG)-deficient keratinocyte model of Atopic Dermatitis^19^. We created a FLG-deficient cell line via lentiviral particles targeting FLG (named thereafter shFLG cells), and a control line transfected with a lentivirus inserting GFP in order to further distinguish between FLG positive and negative populations within the model (shLUC cells) (Supplementary Fig. 5A). Both shFLG and shLUC NHKs demonstrated the correct polygonal morphology within 2D monoculture (Supplementary Fig. 5A, a and b), while only shLUC NHKs displayed green fluorescence as anticipated (Supplementary Fig. 5A, c and d).

**Figure 5:**
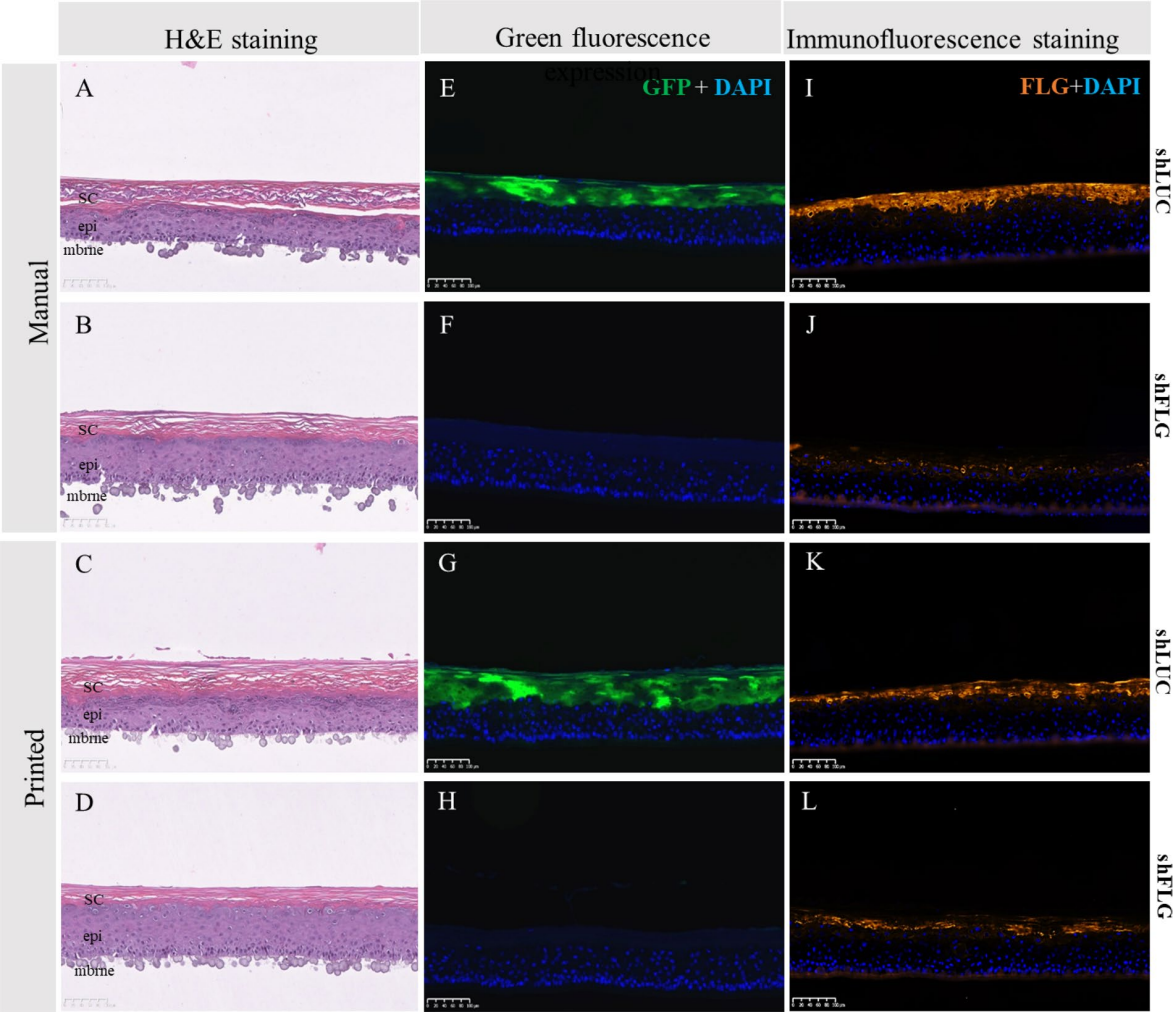
Histological examination of skin models reconstructed via manual seeding or bioprinting with shLUC or shFLG cells. (**A**) H&E sections of shLUC reconstructed samples manually seeded show a well-organized epidermal architecture, while (**B**) shFLG reconstructed samples demonstrate a pronounced hypogranulosis, consistent with FLG down-regulated skin. These data are consistent with those obtained via bioprinting (**C**,**D**). Manually seeded shLUC models expressed high levels of (**E**) GFP, which is not presented in shFLG samples (**F**). Manually seeded samples are indistinguishable from their bioprinted counterparts (**G**,**H**). Lastly, manually printed shLUC models highly express FLG (**I**), in contrast to samples reconstructed with shFLG NHK (**J**). There is no discernible difference in expression in models reconstructed using bioprinting (**K**,**J**). Images representative of 8 separate samples. *SC* stratum corneum, *epi* epidermis, *mbrne* polycarbonate membrane support.

To evaluate the impact of the bioprinting process and shFLG and shLUC cell phenotypes on epidermal differentiation, we created 3D reconstructed epidermal models using either conventional manual cell seeding or bioprinting of both NHK populations on a single polycarbonate membrane. As shLUC and shFLG NHK have different doubling times of 17.7 h and 22 h, respectively, we adjusted their initial seeding concentrations in order to obtain homogenous epidermal proliferation and differentiation (Supplementary Fig. 5B). After 14 days of culture we correlated tissue histology with GPF and filaggrin expression, finding both manually seeded or printed shLUC epidermis were fully differentiated (Fig. 5A,C), positive for GFP (Fig. 5E,G) and expressed FLG in spinous, granular and stratum corneum layers as anticipated (Fig. 5I,K). Conversely, shFLG samples displayed a characteristic reduction of the granular layer while basal and spinous layers were unaffected (Fig. 5B,D), consistent with literature reports^19^. Similarly, shFLG samples lacked GFP expression (Fig. 5F,H) while exhibiting a pronounced reduction of FLG expression as expected of lentivirus-transfected FLG knockdown cells (Fig. 5J,L). Immunofluorescence staining demonstrated the expression and localization of main epidermal markers K14, K10, TGM1 and LOR to be equivalent between printed and manually seeded shLUC and shFLG samples, with homogenous expression and similarly no difference between shLUC and shFLG reconstructions (Supplementary Fig. 6A). We verified the expression of FLG via western blot in our printed shLUC and shFLG epidermis, confirming the downregulation of FLG in shFLG but not shLUC samples (Supplementary Fig. 6B). These results demonstrate that both cell populations formed well-stratified epidermal reconstructions phenotypically consistent with literature reports, and that bioprinting has no deleterious effect on the reconstructed model.

### Patterned shLUC and shFLG human keratinocytes keep their designs over the culture period and form fully differentiated patterned epidermises

**Figure 6:**
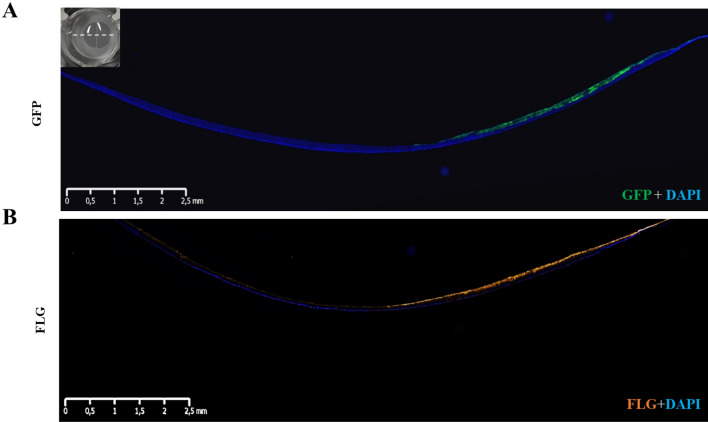
Expression of FLG and GFP in the reconstructed epidermis created via patterned bioprinting with shLUC and shFLG cell populations. (**A**) GFP (green) and (**B**) FLG expression is only present on the shLUC half of the reconstructed skin. Images representative of 8 samples.

As shFLG and shLUC epidermal reconstructions were possible and kept their associated phenotypes, we next bioprinted each population within the same insert to form 2 semi-circles, each printed with either shFLG or shLUC cells. The shLUC half of the model demonstrated strong co-localization of GFP and FLG expression as anticipated, while the half printed with shFLG cells lacked GFP expression with no discernible FLG expression, demonstrating the model to be accurately printed and stable after 14 days in culture (Fig. 6A,B, Supplementary Fig. 7A).

**Figure 7:**
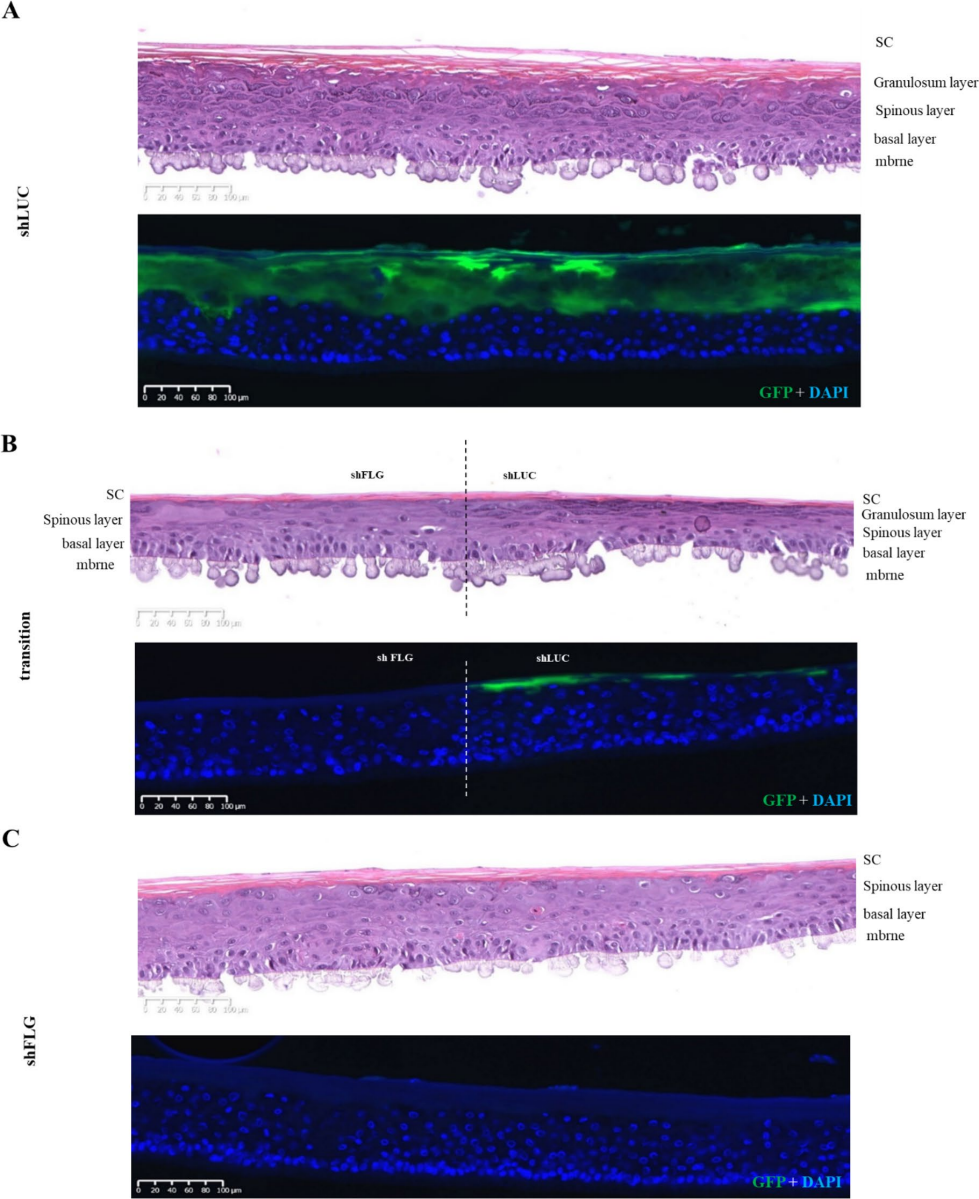
Histological analysis and GFP expression in shLUC and shFLG halves of pattern bioprinted reconstructed skin. (**A**) The suLUC half of the patterned model is histologically normal and shows high levels of GFP staining as anticipated, which abruptly ceases at the border with the shFLG printed half (**B**). (**C**) The shFLG printed half of the model demonstrates a lack of keratohyalin granules in H&E staining and no GFP signal, demonstrating the model to be accurately printed and stable with no migration of cell populations after 14 days in culture. Images representative of 8 individual samples.

The ShLUC half of the reconstructed epidermis presents a normal differentiation with a clear granular layer and presence high FLG expression (Fig. 7A,B), whereas shFLG printed half demonstrate a lack of keratohyalin granules and down-regulation of FLG as previously observed (Fig. 7C, Supplementary Fig. 7A). GFP and FLG expression patterns demonstrate a clear demarcation between shFLG and shLUC printed sections, indicating the cell populations do not stray from their original printed region. Furthermore, the gap between the populations creating during the printing was organically closed by the keratinocyte proliferation over the culture period, resulting in a seamless epidermis composed of the two subpopulations (Fig. 7B). Immunostaining of major epidermal markers was consistent across both shLUC and shFLG printed halves of the reconstruction (Supplementary Fig. 7B). Collectively, these data demonstrate that each printed compartment retains their anticipated phenotype with a clear separation between the printed subpopulations.

## Discussion

Within the present study, we demonstrate how separate populations of NHK can be bioprinted in a precise and controlled way to create accurate patterns, retain their localization over the culture period and form a stable, compartmentalized single sample. This approach allows for complex modelling of skin, and likely increases evaluation study statistical power by enabling the use of pairwise statistics and resulting in a reduced number of models required per study.

Skin is a dynamic living tissue with a high degree of cellular and structural heterogeneity^1,2^. Accurate reproduction of this heterogeneity within in vitro reconstructed skin models is an absolute requirement for translation to both knowledge studies and in vitro evaluation predictability. Traditional manual seeding reconstruction techniques are hampered by cell placement insufficient of the precision required to create more complex and therefore physiologically relevant models of skin heterogeneity. Cellular patterning has already previously examined in 2D and 3D models, but the techniques used within the present study facilitate a higher degree of viability and epidermal stability than previous reports^14,23^. In a pioneering study, Min and colleagues created a full thickness skin model with a controlled deposition of melanocytes to mimic skin blemishes^24^. This approach was not without difficultly however, as after air–liquid interface culture the melanocytes spread across the epidermis rather than restricted to the basal layer as seen within native human skin^6^. A strength of our study is that native skin architecture is well maintained, with a well-stratified differentiation of keratinocytes, from the basal layer up to the stratum corneum^25^, which also result in a model that is highly stable during the culture period.

To better approximate complex in vivo skin, we have validated proof-of-concept-patterning approaches with two different populations of NHK, following two different designs. Utilizing a semi-circle design allows for evaluation of two phenotypically distinct populations in a single insert, which can easily be separated into two halves for analysis. The bioprinted concentric rings approach resembles a dermatological spot, resulting in a more accurate in vivo approximation of spots on skin. Cell printed with either technique showed the correct differentiation of all epidermal layers for their phenotype and express key epidermal markers consistent with published data^21,22^, demonstrating the stability of the pattern printed approach.

The FLG down-regulated NHK epidermal model is a key model for the study of AD and IV, which are widespread in industrialized countries and among the most common human epidermal disorders^18,19,26–28^. We chose to examine this model based on clinical importance, applicability to reconstructed skin approaches, and that the patterned approach is highly suited to examining the edges of AD and IV lesions. Consistent with published data, we note a characteristic dramatic decrease of keratohyalin granules, demonstrating our reconstruction to be consistent with those published previously^29^. Of note, we did not observe difference for intermediate and final differentiation markers as K10 or LOR in our shFLG models compared to controls, consistent with the results of the FLG down-regulated epidermal model of Mildner and co-workers, but contradictory to the model of Pendaries and colleagues^18,19^. It is interesting that the presence of shFLG cells within our patterned AD model did not influence the differentiation of the control shLUC cells, with all layers of the epidermis and epidermal marker expression being normal, while shFLG printed sections showed down-regulation of the FLG protein associated with attended hypogranulosis exclusively in the shFLG section. Our results demonstrate that these cells can both exist in separate sides of a single insert, a prerequisite for practical use of patterned skin bioprinting.

The compartmentalization of several cellular types on a same reconstruction, including functionalized cells modified by genome editing, allows for greater understanding of physiological and pathophysiological mechanisms of human skin. The principles described within this paper are likely to be applicable to any model using keratinocytes as the constituent cells that requires a pattern design. For instance, patterned epidermal models may have applications for knowledge studies of skin diseases, particularly skin cancers of keratinocyte origin. Such a model would be valuable to examine preventive treatment for photoaging associated with actinic keratosis, whereby the epidermis presents patches of p53 mutated NHKs that may progress to invasive squamous cell carcinoma^15^*.* The patterning approach is similarly applicable for raw material evaluation safety and efficacy studies. Skin from different parts of the body could be pattern onto a single model (for example to model skin of the face or arm), with cells derived from donors of different ethnicities or ages in order to study their phenotypic differences. Such heterogenous models may be used to examine contrasting responses to environmental or chemical stresses. The shFLG model could be of interest to study such as microbiota interactions, impact of UV associated with humidity and temperature, or pollutants to better understand some mechanisms associated to AD and IV^19,29–31^. The precision of bioprinting also offers the potential to increase the complexity of epidermal models by integrating langerhans cells or melanocytes, or by printing a fibroblast-populated dermal compartment with immune cells as T helper cells to study AD pathology^30,32,33^*.* Patterning melanocytes, langerhans cells or creating complex dermal compartment reconstructions will need adaptations of protocols and the materials however, as they all present increased cellular mobility, facilitating inter- and intra-compartmental migration.

A particular strength of the patterning approach is that the dual population model can be used to test a raw material or drug of interest on both sides simultaneously. This technique creates the opportunity to use paired statistical approaches, reducing the inter-section variability and thus increasing the statistical power the evaluation studies. Similarly, the pairwise approach of having two conditions on a single sample facilitates a dramatic reduction in the total amount of samples required to achieve meaningful results within a study, leading to reduce study costs and higher result reliability. To get high reproducibility in the cell deposition and to be able to process our samples always the same way after culture, we have patented an adaptor for insert. This adaptor blocks inserts during printing and culture with a precise orientation, which is essential for sample pooling and/or comparison regarding in vitro evaluations and knowledge.

In summary, by integrating different cellular populations in a same model using a high precision patterned bioprinting approach we open the possibility of creating heterogeneous yet stable reconstructive skin models more reflective of native human skin. The patterned human epidermis model described within this study is the first proof-of-concept description that such a bioprinting based-strategy can create a viable and stable NHK patterned reconstructed epidermis. This innovation in tissue engineering offers potential for basic research into skin pathology using transfected keratinocytes to model skin conditions, and provides a stable and robust basis of evaluation studies with potential for improved statistical power and reduced tissue use.

## Materials and methods

### Keratinocyte culture and preparation

Primary NHK were collected from healthy foreskin originating from surgical waste of consenting subjects. The cells were cultured on a feeder layer of Mitomycin C-treated 3T3 fibroblasts and in presence of ROCK Inhibitor (Y-27632, Sigma-Aldrich, USA) in an incubator at 37 °C and in a humidified atmosphere containing 5% of CO_2_ in NHK growth media (Episkin, France). Cells were detached using 0.05% Trypsin–EDTA (1×) (Gibco, USA) after phosphate buffered saline (PBS, ThermoFisher, USA) washing, and then suspended in a SGM medium (Episkin, France) at concentrations ready to use for manual seeding or printing.

### Mitomycin C treatment for feeder production

Murine 3T3 fibroblasts were cultured in DMEM (Gibco, USA) enriched with 10% of Bovine calf serum defined/supplemented (BCS, HyClone, USA) medium for 5 days, at an initial density of 1.5 × 10^6^ cells, in an incubator at 37 °C and in a humidified atmosphere containing 5% of CO_2_, with a medium change every two days. When 3T3 covered 70–80% of the flask area, they were treated with a solution of 0.5 mg/ml of Mitomycin C (Sigma, USA) and Dulbecco's phosphate-buffered saline enriched with calcium and magnesium (PBS+, ThermoFisher, USA) solution for two hours in a 37 °C/5% CO_2_ incubator. Mitomycin C-treated 3T3 were washed with phosphate buffered saline (PBS, ThermoFisher, USA) and harvested at passage 13 using 0.05% Trypsin EDTA (1×) (Gibco, USA), resuspended in DMSO (Sigma, USA) 7.5% diluted in DMEM + 15% of BCS and homogenously distributed in cryopreserved vial at 1 × 10^7^ cells per vial and stored in liquid nitrogen for future use.

### Normal human keratinocytes GFP transduction

Primary NHK were collected from foreskin of young healthy subjects under plastic surgery were purchased from Alphenyx (France), or Biopredic (France), both of which are accredited by the French Minister of Research. Informed consent was obtained from all subjects or from a parent and/or legal guardian for donors under 18 years of age. All samples of skin cells collected NHK were transduced at multiplicity of infection (MOI) of 5 with highly purified lentiviral particles (Flash Therapeutics, Toulouse, France) with either a Green Fluorescent Protein (GFP), a control luciferase targeting shRNA (shLuc) or a filaggrin targeting shRNA (shFLG: 5′-TAGTTTGGTGGTAGCTTATTT-3′), with 4 μg/ml of polybrene (SIGMA, USA). After 18 h of incubation, plates were rinsed in PBS (ThermoFisher, USA) and cultured in NHK Growth medium (Episkin, Lyon, France). shFLG and GFP cells were then selected with puromycin (InvivoGen, USA) at 5 µg/ml in NHK Growth medium (Episkin, France) for 48 h.

**Table:** 

Name	Vector description
shLUC	rLV.H1.shLuc.EF1.GFP
shFLG	rLV.U6.sh4FLG.hPGK.PURO
GFP	rLV.EF1.EGFP.hPGK.PURO

### Doubling time

Transduced cells were trypsinized and counted 5 days after plating to evaluate the population doubling time compared to non-transduced keratinocytes using the following equation:

Doubling time = (log(2) × Duration of culture/(log(final concentration) − log(initial concentration)).

### Inkjet bioprinting

Keratinocyte suspensions were transferred into sterile UV shielding bioprinting cartridges (Nordson EFD, USA) and loaded onto the bioprinter along with an additional mechanical cell mixer device (RegenHU, Switzerland). Normal and transduced NHK were seeded seperately at an initial cellular density of 200 × 10^3^ up to 300 × 10^3^ NHK/0.5 cm^2^, directly onto air-lifted polycarbonate culture inserts (Episkin, Lyon, France) blocked on 6 well-plates by an internal adaptor (French patent application No FR3067040, filed on 2nd of June 2017). Cellular suspensions were extruded through a CF300 ID (inner diameter) = 0.15 mm/S (stroke) = 0.1 mm jetting piezoelectric microvalve inkjet printhead nozzle (RegenHU, Switzerland) at a pneumatic pressure of 0.010 MPa into semi-circles and concentric rings patterns. Single layers were printed for both designs at 800 μm printing resolution (distances between the droplet dispensing) and using a valve opening and closing duration of 2000 µs. After bioprinting, the reconstructed epidermis models were cultured in an incubator (37 °C/5% CO_2_) at air-lift interphase for the entirety of the reconstruction period. For WT/WTGFP, experiments were carried out two times and for shFLG/shLUC, experiments were carried out 3 times.

### Manual cell deposition

Normal and transduced NHK were manually seeded at an initial cellular density of between 200 × 10^3^ to 300 × 10^5^ NHK/0.5 cm^2^, then directly onto air-lifted polycarbonate culture inserts (Episkin, France) and blocked on 6 well-plates by an internal adaptor. The models were then cultured in an incubator (37 °C/5% CO_2_) at air-lift interphase for the entirety of the reconstruction period. For WT/WTGFP experiments were carried out 2 times, while shFLG/shLUC, experiments were done 3 times.

### Patterning designs

BioCAD (RegenHU, Switzerland) bioprinting software was used to draw designs in all bioprinting studies. Two patterns were generated; semi-circles and concentric rings. The concentric rings design is composed of two compartments; the inner circle and the outer circle. The inner circle includes an area of 0.38 cm^2^ and the outer circle an area of 0.98 cm^2^. Both are composed of consecutive circles separated by 500 µm each. Inner and outer circles are separated by a 1.5 mm space. The semi-circles design is also composed of two mirrored semi-circle compartments separated by a 1.5 mm space. Each semi-circle has an area of 0.56 cm^2^ and are composed of vertical lines each separated by 800 µm.

### Human epidermis reconstruction

Reconstructed human epidermis (RHE) were cultured for 14 days with SGM medium at air–liquid interface and media was replaced every other day.

### Sample preparation and immunostaining on frozen sections

Mature epidermis constructs were collected and cut in half, perpendicularly to the compartmentalization imposed by the pattern. Half was embedded in optimal cutting temperature (OCT) compound (Tissue Tek, Sakura, Japan) in cryomold biopsy molds (Fisher USA) and frozen in a dry ice/100% ethanol bath. The samples were then sent to a subcontractor, (ABS, Saint-Herblain, France) in order to perform immunostaining. The samples were cut with a cryostat at − 20 °C (block and chamber support temperature) to create 7 µm thick sections. The frozen sections were soaked 10 min in acetone at − 20 °C, then washed in 1 × PBS for 5 min at room temperature. The samples were blocked in 1 × PBS/ Bovine Serum Albumin (BSA, Sigma, USA) 3%/Normal Goat Serum (NGS, Diagomics, France) 10% solution for 30 min at room temperature. Primary antibodies for Keratin 10 (DAKO, Agilent, USA, 1/100 dilution), Keratin 14 (Abcam, GB, 1/1000 dilution), Loricrin (Ozyme, France, 1/500 dilution), Transglumatinase 1 (Santa Cruz, USA, 1/400 dilution and Novus Biological, USA, 1/500 dilution) were incubated over night at 4 °C. Filaggrin (Santa Cruz, USA, 1/200 dilution) was stained for 30 min at room temperature in an Antibody diluent (Diagomics, France). Then, sections were rinsed 3 times with 1 × PBS for 5 min at room temperature. Secondary antibodies (Goat anti-mouse AF555, Life Technologies, 1/500 or Goat anti-rabbit AF555 Life technologies 1/500) were incubated for 1 h at room temperature in a dark chamber. Then, sections were rinsed 3 times with 1 × PBS for 5 min at room temperature. Slides were then mounted in Prolong Gold antifade reagent with DAPI (Life Technologies, USA). Immunofluorescence and fluorescence microscopy scans were achieved with a Nanozoomer S60 (Hamamatsu, Japan) and analyzed with NDP view software.

### Sample preparation and histological staining on paraffin sections

Unfixed mature printed epidermis constructs were collected and cut in half, perpendicularly to the compartmentalization imposed by the pattern. Half is placed in a histological cassette between 2 foam pads (both Dutscher, Belgium) prewet in 4% paraformaldehyde (w/v) buffered at pH 6.9 (Carlo Erba Reagents, France) and incubated at 4 °C. The samples were then sent to a subcontractor (Atlantic Bone Screen, France) in order to perform H&E staining. The paraformaldehyde fixed samples are dehydrated by a succession of ethanol 70%, ethanol 100% and xylene baths and embedded into a paraffin block. Samples are cut with a microtome at room temperature (block and chamber support temperature) to 5 µm thickness. Paraffin embedded samples underwent deparaffinization (ethanol, xylene and distilled water baths) before being placed onto SuperFrost Plus (ThermoFisher, USA) microscope. After being deparaffinized and rehydrated, sections are immersed in Hematoxylin solution (ThermoFisher, USA) for 2 min. Then, they were washed and immersed 2 min in bluing solution (ThermoFisher, USA) and washed again. The samples were then stained 6 min with 1% Eosin Y solution (ThermoFisher, USA). After the washing step, sections are dehydrated with ethanol and xylene, and mounted with Coverquick 2000 Q path mounting medium (VWR). Sample scans were achieved with a Nanozoomer S60 (Hamamatsu, Japan) and analyzed with the NDP view software.

### FLG quantification by Western Blot

RHE model samples were homogenized, in a lysis buffer with 300 µl of RIPA containing a cocktail of protease inhibitors (Roche, Switzerland), pepstatine (Roche, Switzerland) and steel balls, 2 × 3 min at 30 Hz. Lysates were clarified by centrifugation at 10,000*g* at 4 °C. Protein determination was performed on subsequent soluble protein supernatants using a BCA assay (Thermo Scientific, USA) according to the manufacturer’s instructions. Eight µg aliquots of soluble protein extracts from samples were separated on TGX stain free 8–16% gradient SDS-PAGE gels (Bio-Rad, USA). Proteins were then transferred to nitrocellulose membranes (Bio-Rad) under standard conditions 7 min at 25 V. Membranes were incubated 2 × 15 min with TBS Tween/1% Blotting Grade Blocker (Bio-Rad, USA). Then primary antibodies were incubated overnight at 4 °C, followed by anti-HRP conjugated secondary antibody (Bio-Rad, USA) in TBS Tween/1% Blotting Grade Blocker (Bio-Rad, USA) 1.5 h at room temperature. Protein bands were revealed by ECL Prime (GE Healthcare, USA) and visualized on ChemiDoc MP (Chemi Hi Resolution).

**Table:** 

Protein	Reference	Dilution	Anti-species	MW
Actin	sc47778 Santa Cruz	1/1000	Mouse	43 kDa
Filaggrin	sc66192 Santa Cruz	1/200	Mouse	38 kDa

## Supplementary Information

Supplementary Information.
