# Author Correction: Utilization of patterned bioprinting for heterogeneous and physiologically representative reconstructed epidermal skin models

**DOI:** 10.1038/s41598-021-97269-5

**Published:** 2021-09-01

**Authors:** Sabrina Madiedo-Podvrsan, Jean-Philippe Belaïdi, Stephanie Desbouis, Lucie Simonetti, Youcef Ben-Khalifa, Christine Collin-Djangone, Jérémie Soeur, Maïté Rielland

**Affiliations:** L’Oréal Research and Innovation, Aulnay-sous-Bois, France

Correction to: *Scientific Reports* https://doi.org/10.1038/s41598-021-85553-3, published online 18 March 2018

Christine Collin-Djangone was omitted from the author list in the original version of this Article.

The Author Contributions section now reads:

“S.M.P.: performed all experiments, methodology. J.P.B.: co-performed all experiments, methodology. S.D., L.S., Y.B.K.: co-performed transduction and western blot experiments. C.C.D.: performed experiment on plasmid constructions and validations. J.S.: funding acquisition, project administration, reviewing and editing original draft. M.R.: project administration, supervision, writing original draft preparation, prepared all figures.”

The original Article and accompanying Supplementary Information file have been corrected.

