# Supplementary material for: Utilization of patterned bioprinting for heterogeneous and physiologically representative reconstructed epidermal skin models
Source: Sci Rep. 2021 Mar 18;11:6217. doi: 10.1038/s41598-021-85553-3 (PMC7973417; doi:10.1038/s41598-021-85553-3)
Supplement: Supplementary file 1 — Supplementary Information. [file 41598_2021_85553_MOESM1_ESM.docx]

**Utilization of patterned bioprinting for heterogeneous & physiologically representative reconstructed epidermal skin models**

**Sabrina Madiedo-Podvrsan^a1^ & Jean-Philippe Belaïdi^a1^, Stephanie Desbouis^a^, Lucie Simonetti^a^, Youcef Ben-Khalifa^a^, Christine Colin-Djangone ^a^, Jérémie Soeur^a*^ & Maïté Rielland^a*^**

1. *L’Oréal Research and Innovation, Aulnay-sous-Bois, France.*

*^1^ These authors contributed equally to this work*

** Correspondence to* [*jeremie.soeur@rd.loreal.com*](mailto:jeremie.soeur@rd.loreal.com) *or* [*maite.rielland@rd.loreal.com*](mailto:maite.rielland@rd.loreal.com)*. These authors contributed equally to this work and are corresponding authors*

***Supplemental data:***


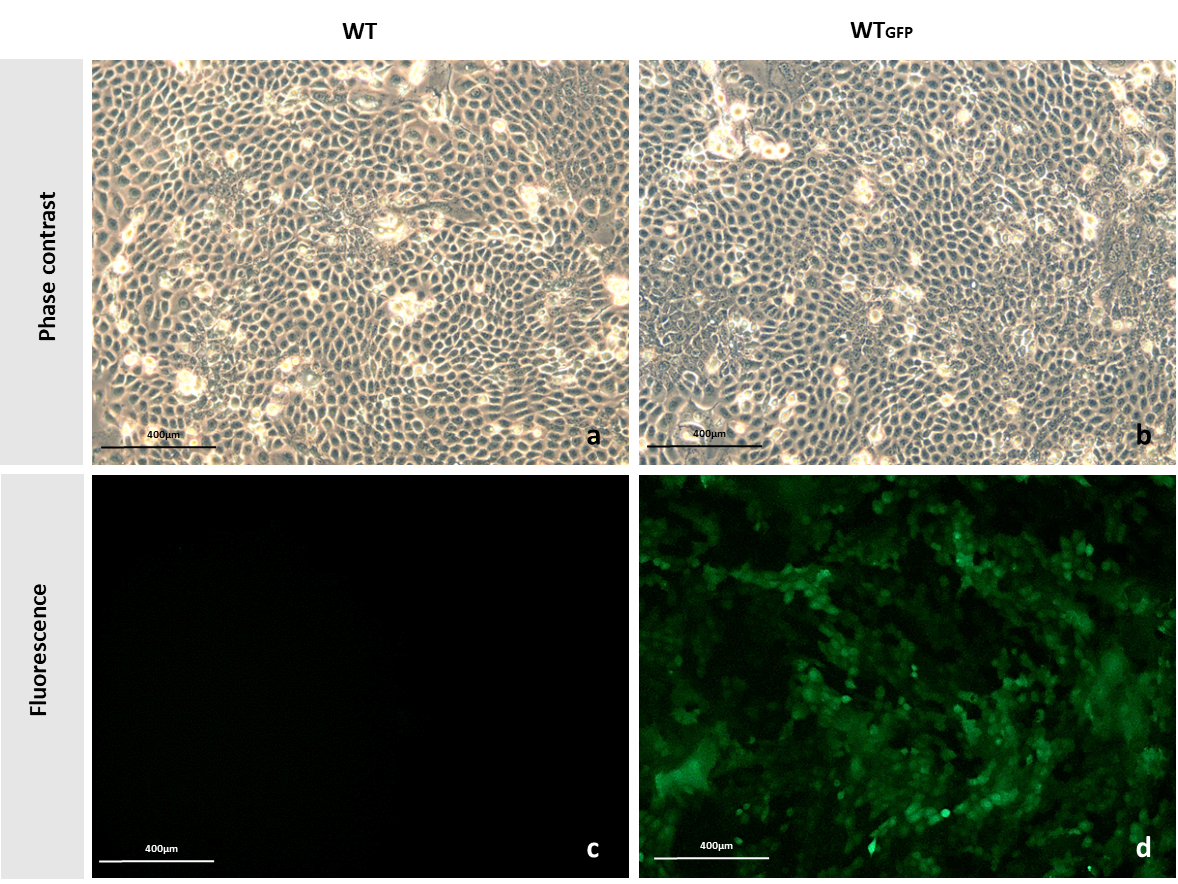


**Supplemental Figure 1.** Normal human keratinocytes in our study formed two subpopulations; one untouched population WT (a and c), and the other transduced with GFP: WT-GFP (b and d). Phase-contrast imaging of WT NHKs (a) and WT-GFP NHKs (b) and their corresponding fluorescence imaging for WT NHKs (c) and WT-GFP NHKs (d) after 120h of culture, showing correct morphologies for WT and WT-GFP, and green fluorescence expression in the WT-GFP population.


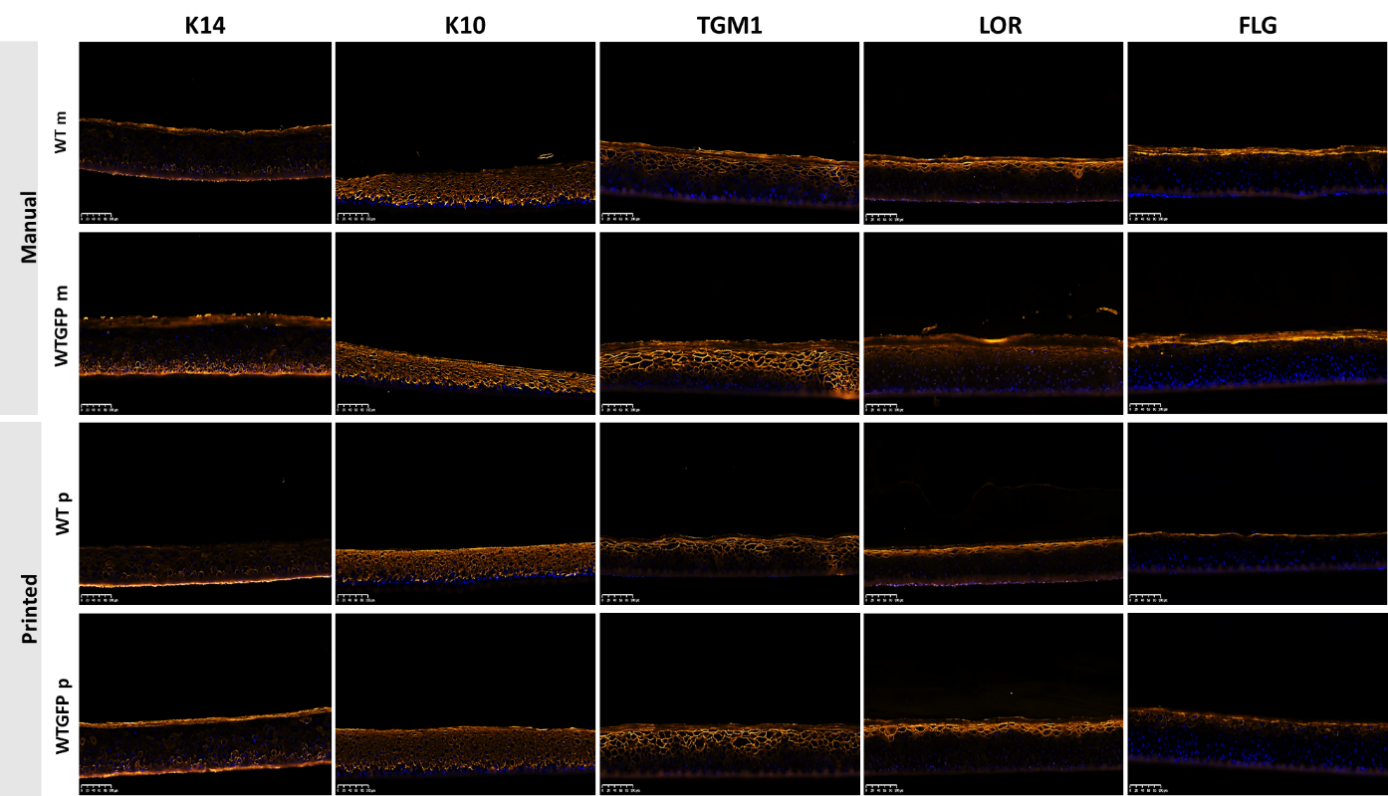


**Supplemental Figure 2.** Immunofluorescence characterization of key epidermal markers in manual and printed reconstructed epidermal models derived from WT or WT-GFP NHKs. Main epidermal markers as Keratin 14 (K14) for the basal layer; Keratin 10 (K10) for the spinous and granular layers, Transglutaminase 1 (TGM1), Loricrin (LOR) and Filaggrin (FLG) for final differentiation layer were analyzed via immunofluorescence, with both manually and printed reconstructed models correctly express main markers of the epidermis. The marker expression for WT or WT-GFP NHK-derived models were similarly equivalent. Markers of interest are immunostained in gold and nuclei are counterstained with DAPI in blue.

*
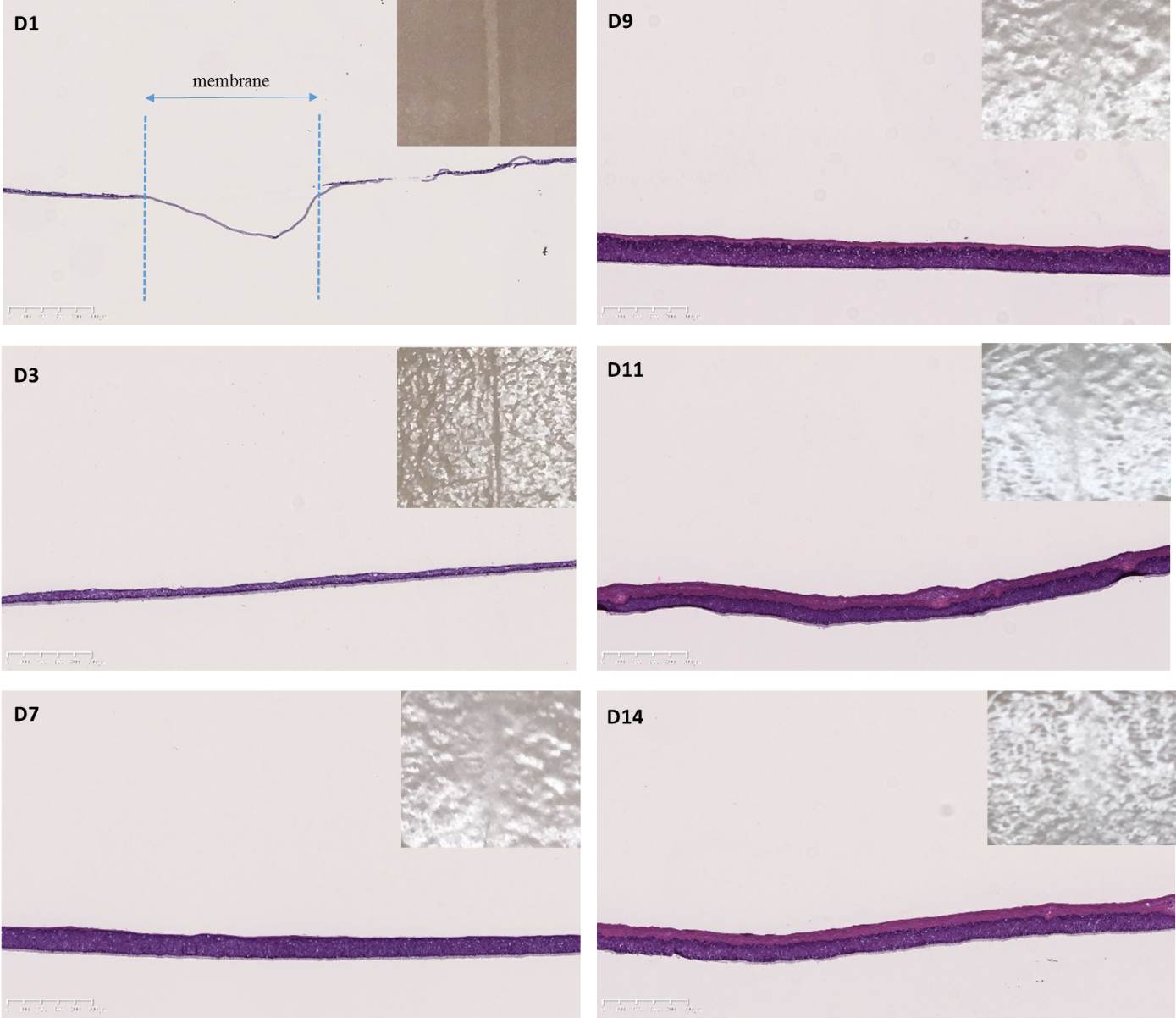
*

**Supplemental Figure 3.** H&E staining on patterned models showing the colonization of WTGFP and WT NHKs at day 1 (D1), day 3 (D3), day 7 (D7), day 9 (D9), day 11 (D11) and day 14 (D14) and their associated macroscopic pictures, proving that NHKs have populated the separation distance between compartment from D3.


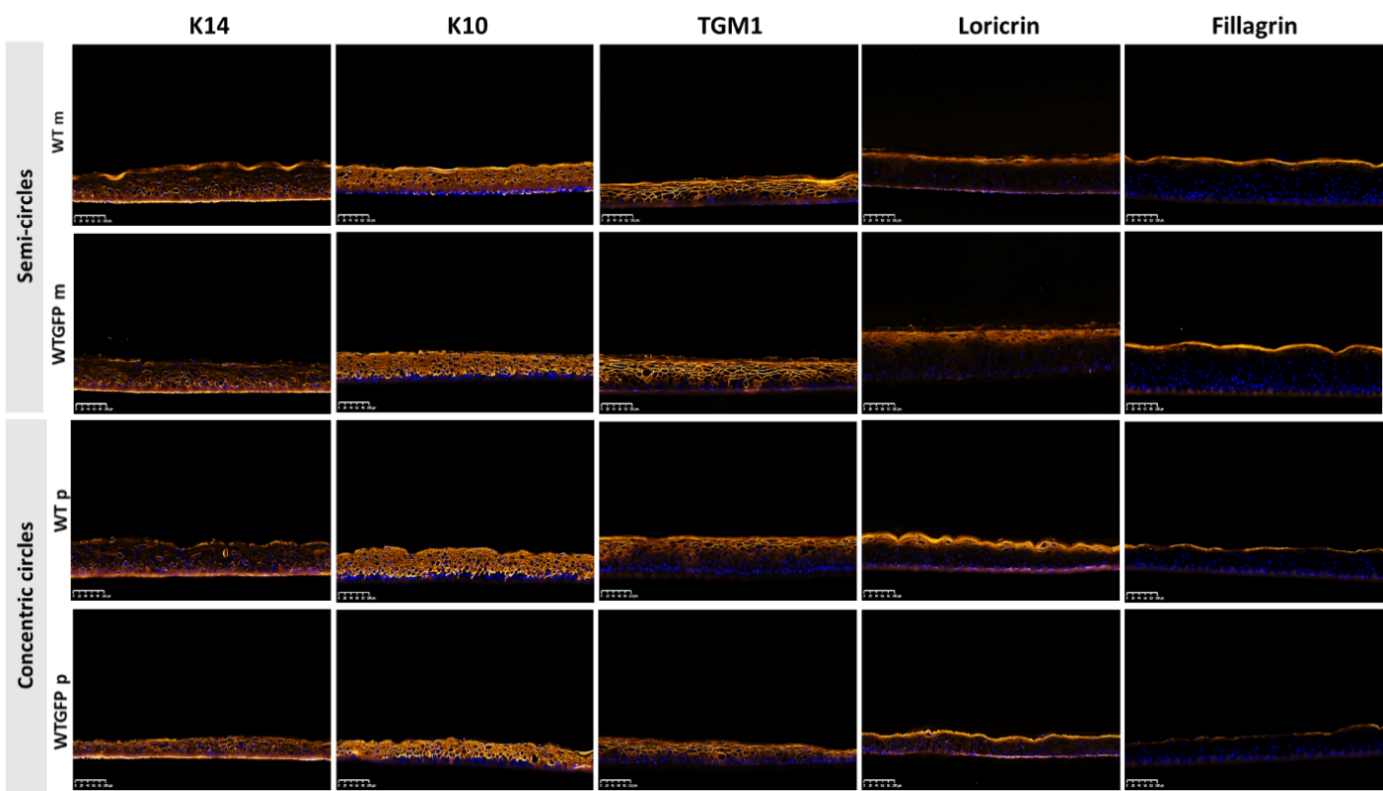


**Supplemental Figure 4.** Characterization by immunofluorescence staining of semi-circle and concentric patterned reconstructed epidermal models obtained WT or WT-GFP NHKs. WT and WT-GFP parts of the patterned samples correctly express main markers of the epidermis, irrespective of pattern printed or cell phenotype. Markers of interest are immunostained in gold and nuclei counterstained with DAPI in blue.


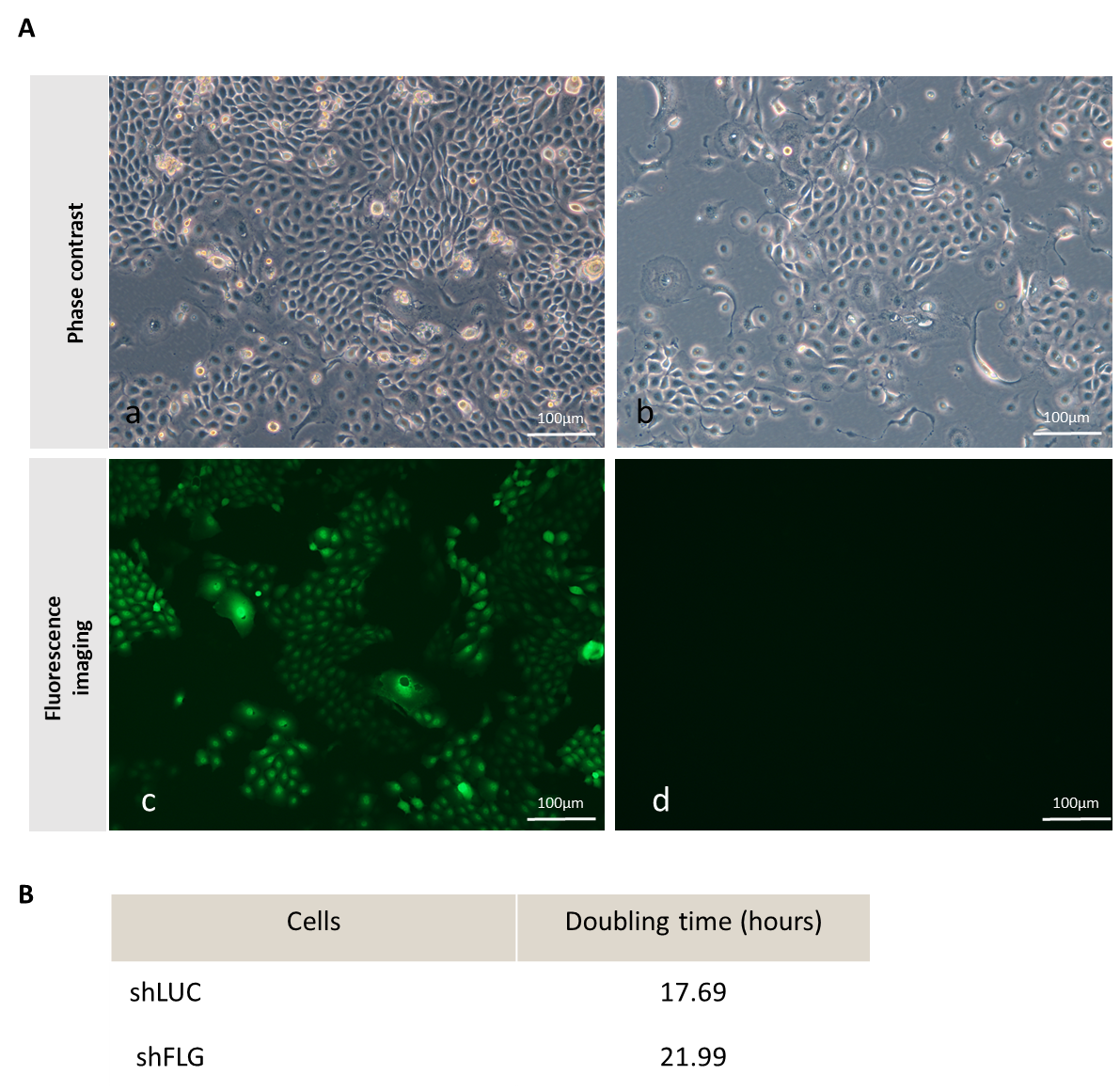


**Supplemental Figure 5**. Lentiviral GFP transduction of NEK. **A-** Cells were transduced either with a shLUC to express GFP (**a & c**) or shFLG to knock-down FLG (**b & d**). Images were taken after 120 hrs of culture, with both groups showing regular cell morphology and strong GFP expression for shLUC cells as anticipated. B- doubling time of shLUC and shFLG populations. Cells were counted 5 days after plating


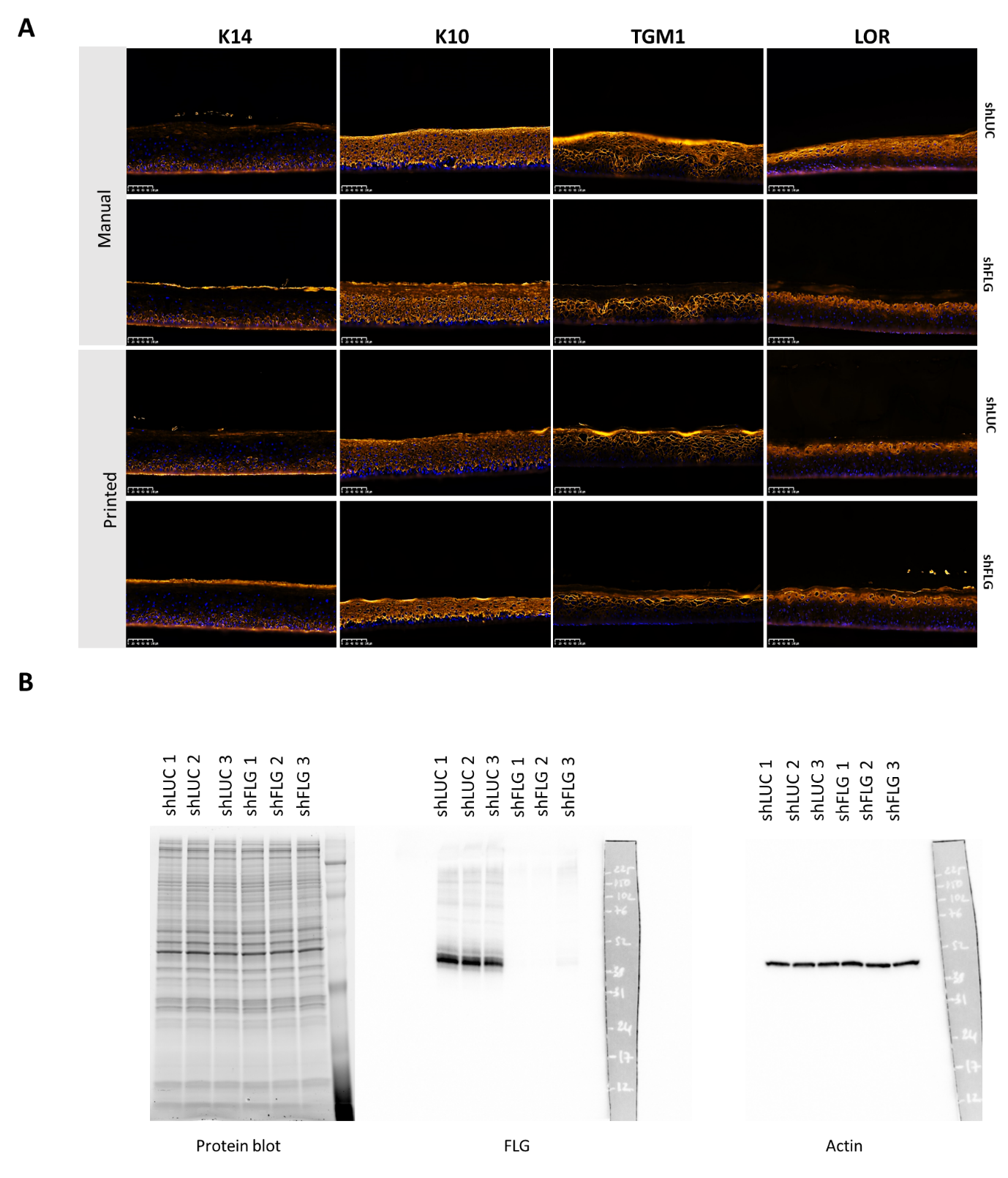


**Supplemental Figure 6**. Characterization of shLUC and shFLG bioprinted patterned skin. (**A**) Characterization key epidermal markers by immunofluorescence staining of shLUC and shFLG reconstructed epidermal models, showing similar staining levels between groups. Markers of interest are immunostained in gold and nuclei are counterstained with DAPI in blue. (**B**) Western Blot analysis for FLG and Actin confirming that FLG is correctly down-regulated in 3 shLFG samples (shFLG1, 2 and 3) compared to 3 shLUC samples (shLUC1, 2 and 3) after 14 days at air-lift interphase.

*
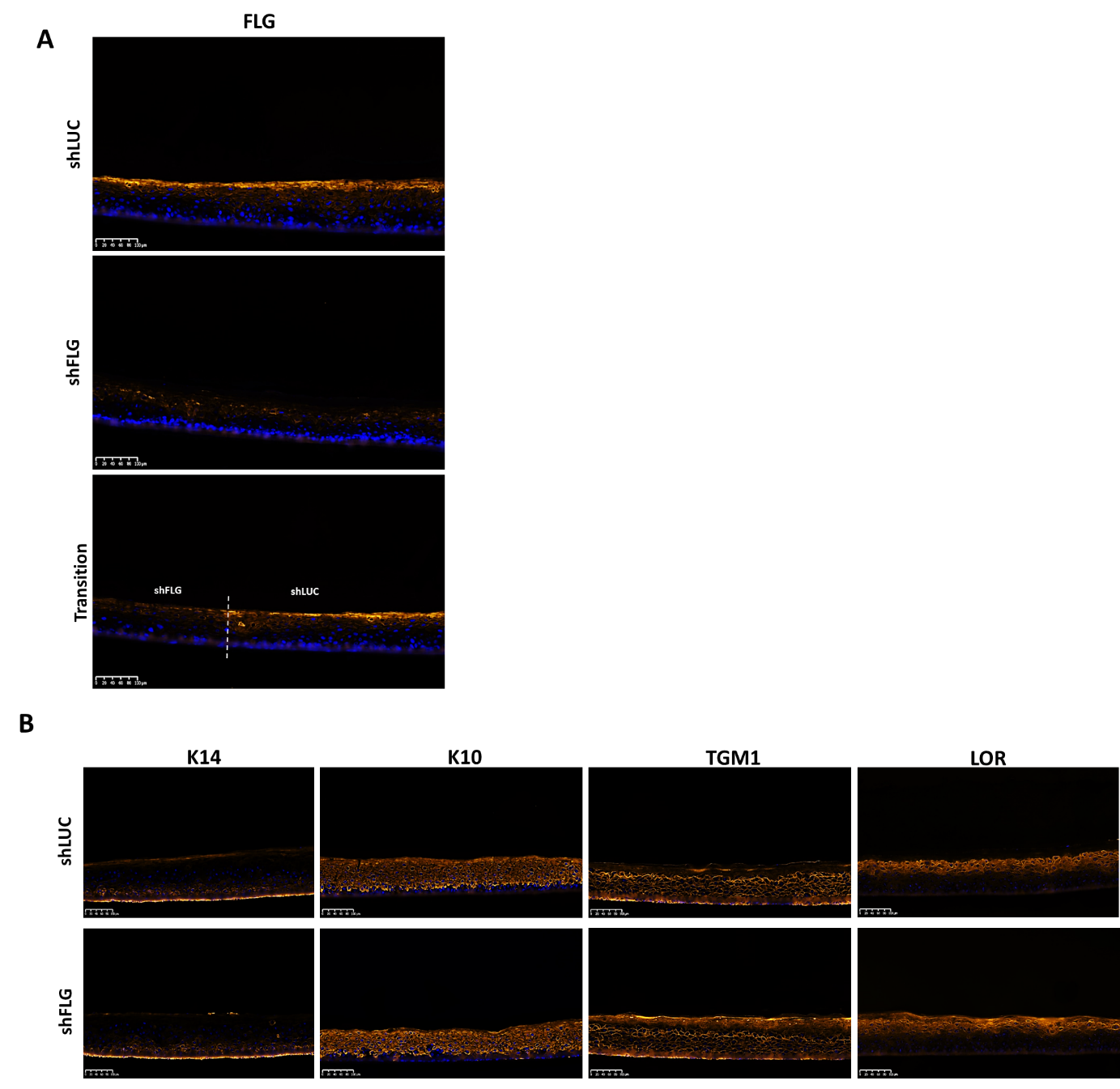
*

Supplemental Figure 7: Immunofluorescence staining of each semi-circle of the shLUC/shFLG printed model epidermal model. (**A**) FLG is highly expressed in the shLUC half of the tissue and poorly expressed in the shFLG side of the tissue, with a sharp demarcation where the 2 halves meet in the middle. (**B**) Key epidermal markers are immunostained in gold and nuclei are counterstained with DAPI in blue, showing consistent expression across shLUC and shFLG halves of the tissue.
